# Preemptive fixation of a jejunal enteral tube extension via novel anchoring system

**DOI:** 10.1055/a-2774-4394

**Published:** 2026-01-20

**Authors:** Jonathan Rozenberg, Rami J. K. Musallam, William F. Abel, Vivek Kesar, Patrick I. Okolo, Varun Kesar

**Affiliations:** 1 6912 Department of Internal Medicine, Virginia Tech Carilion, Roanoke, Virginia, United States; 2 6912 Department of Internal Medicine, Division of Gastroenterology, Virginia Tech Carilion, Roanoke, Virginia, United States; 3 22161 Department of Internal Medicine, Division of Gastroenterology, Stony Brook University Hospital, Stony Brook, New York, United States

**Fig. 1:**
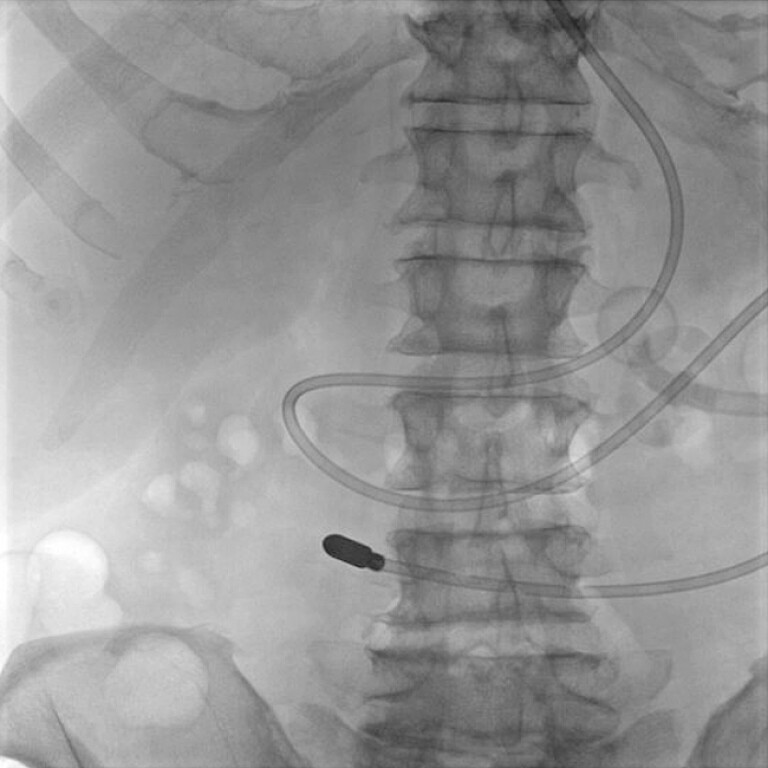
A fluoroscopic image depicting scout imaging of the previously placed percutaneous endoscopic gastrostomy (PEG) tube bumper and a nasojejunal (NJ) tube with its tip in the proximal jejunum, respectively.

**Fig. 2:**
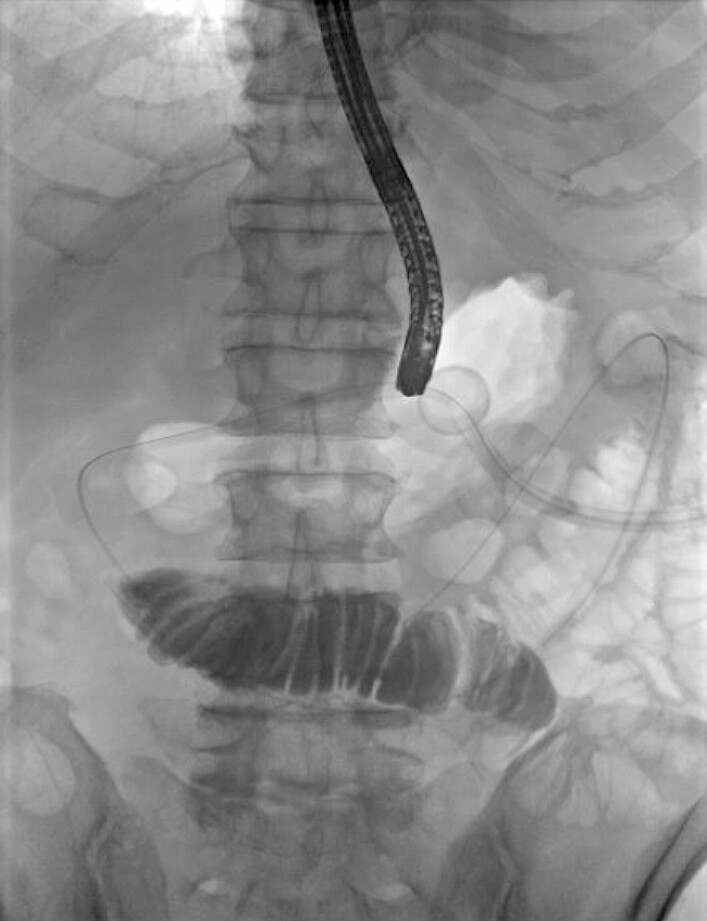
A fluoroscopic image demonstrating successful over the wire jejunal arm extension into the proximal jejunum past the ligament of Treitz.

**Fig. 3:**
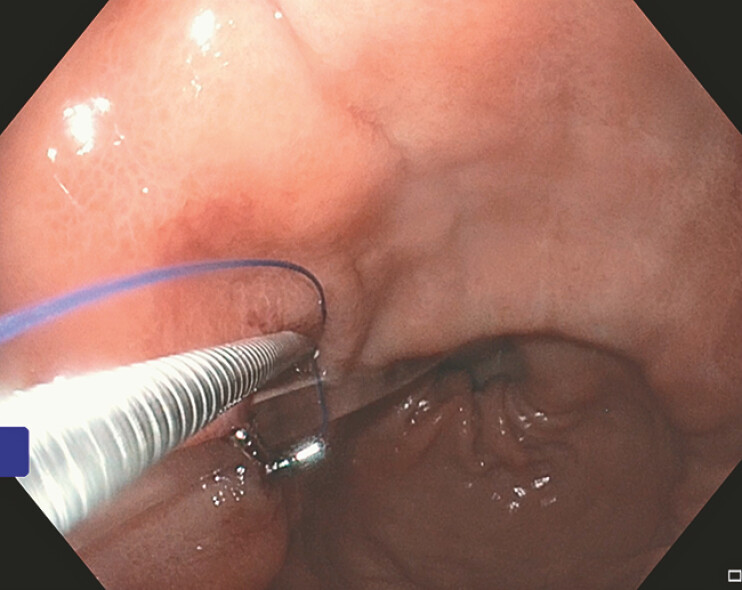
An endoscopic image of HeliX Tack placement, superior to the PEG-jejunum (PEG-J) tube, along the anterior aspect of the proximal gastric body.

**Fig. 4:**
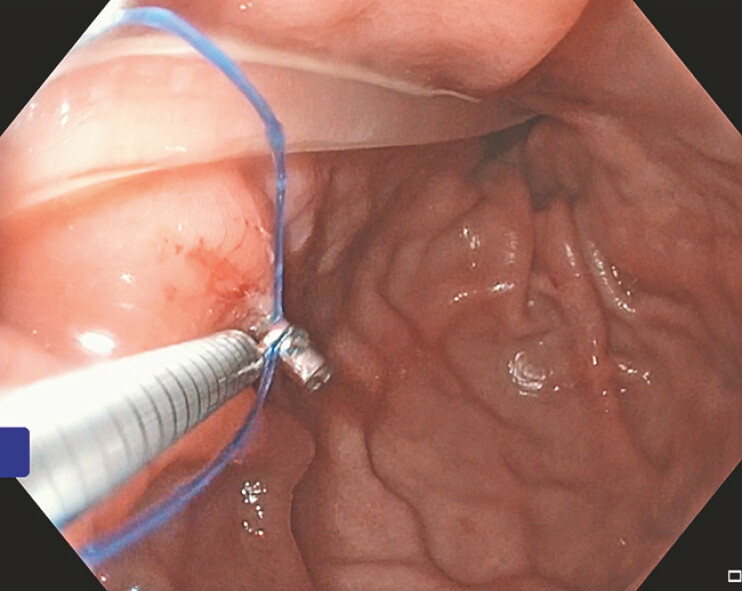
An endoscopic image of HeliX Tack placement, inferior to the PEG-J tube, along the anterior aspect of the proximal gastric body.

**Fig. 5:**
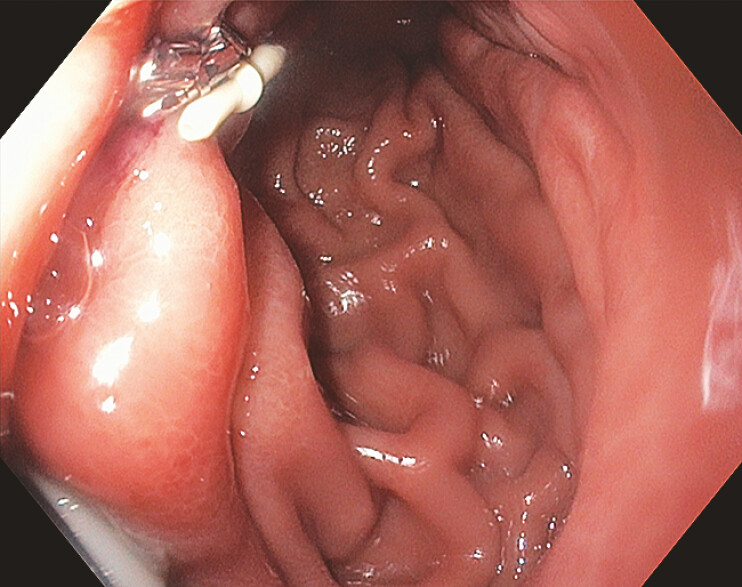
An endoscopic image exhibiting complete PEG-J arm fixation to the proximal aspect of the gastric body via the X-Tack anchoring system.

We present a case of a 76-year-old man with a pertinent past medical history of severe pharyngeal dysphagia status post percutaneous endoscopic gastrostomy (PEG) tube who presented for nasojejunal (NJ) to PEG-jejunostomy (PEG-J) conversion. Two prior attempts at jejunal-arm extension failed secondary to initial proximal positioning of the PEG tube and its consequent migration peri-procedurally. Initial scout films demonstrated the PEG tube bumper and the NJ tip in the proximal jejunum, respectively ( Fig. 1 ). A guidewire was positioned in the jejunum with subsequent NJ tube removal. The jejunal-arm was then extended, over the wire, into the proximal jejunum past the ligament of Treitz ( Fig. 2 ). Once in position, the X-Tack Endoscopic HeliX Tacking System (Boston Scientific; Marlborough, MA, USA) was utilized to secure the jejunal-arm to the proximal aspect of the gastric body ( Fig. 3 , Fig. 4 , Fig. 5 ) for the prevention of jejunal-arm coiling. Thereafter, he tolerated PEG-J feeds with minimal reflux into the venting gastrostomy-arm and was subsequently discharged.

Prophylactic fixation of percutaneous endoscopic gastrostomy jejunal arm extension to the proximal aspect of the gastric body via the X-Tack Endoscopic HeliX Tacking System (Boston Scientific; Marlborough, MA, USA) for the prevention of gastric coiling.Video 1

PEG tubes routinely serve as a first-line medium to deliver enteral nutrition for a prolonged period; however, associated dysfunctions/complications are not uncommon 1 . PEG tube dislodgement has been reported to occur in 0.6–4.0% of cases within 7–10 days of initial placement, and up to 12.8% long-term 2 . Literature studies regarding endoscopic intervention in PEG tube dislodgement mainly consist of case reports/series for the management of recurrent dislodgment(s) 1 2 3 4 5 . Of these, the OverStitch device (Boston Scientific; Marlborough, MA, USA) has been predominantly utilized 1 2 3 4 with a recent case incorporating the X-Tack system 5 . Given the scarce literature pertaining to this topic, both the role of pre-emptive endoscopic suturing in jejunal-arm extension(s) as well as the efficacy of the X-Tack system in such cases is unclear. As such, this case illustrates the successful NJ to PEG-J conversion with precautionary jejunal-arm fixation via the X-Tack Endoscopic HeliX Tacking System ( Video 1 ).

Endoscopy_UCTN_Code_TTT_1AO_2AK
